# Androgen receptor upregulation regulates tau pathology in a female tauopathy mouse model

**DOI:** 10.1002/alz.091965

**Published:** 2025-01-03

**Authors:** Daniel Munoz‐Mayorga, Xinchen Lyu, Yuren Tao, Alexander S. Kauffman, Robert A. Rissman, Xu Chen

**Affiliations:** ^1^ University of California, San Diego, La Jolla, CA USA; ^2^ Alzheimer’s Therapeutic Research Institute, Keck School of Medicine, University of Southern California, San Diego, CA USA; ^3^ Veterans Affairs San Diego Healthcare System, La Jolla, CA USA

## Abstract

**Background:**

According to data from the Alzheimer’s Association, more than two‐thirds of patients living with Alzheimer’s disease (AD) in the United States are women. The interplay between aging and hormone depletion during menopause has been proposed as a leading cause, but the molecular underpinnings of this vulnerability are not fully understood. On the one hand, approaches that seek to supplement estrogens to rescue pre‐menopausal hormonal levels have had contradictory outcomes in clinical trials. On the other hand, androgen effects in women in a neurodegeneration context are dramatically understudied. Furthermore, the number of XX chromosome individuals using androgens for sex‐reassignment purposes has steadily increased. Whether androgens can regulate tau pathology and how is poorly understood, especially in the context of female physiology.

**Method:**

We performed a dihydrotestosterone (DHT) supplementation paradigm in 5‐6 month old P301S female mice and harvested brain tissue at 9 months of age. Pathological and biochemical tau markers, as well as androgen receptor content were evaluated in the hippocampus and cortex.

**Result:**

Here, we report an exacerbation of tau pathology in tauP301S female mice treated with DHT, an androgen receptor (AR)‐specific ligand, in the hippocampus, as evaluated by AT8 and MC‐1 immunostaining. Furthermore, we report upregulation of AR almost exclusively in neurons upon DHT treatment, potentially mediating our observations.

**Conclusion:**

Our results implicate AR activation in females as a potential risk factor for tau pathology. Overall, the AD field will benefit from a more thorough understanding of neurodegeneration in women and transmasculine individuals, facilitating the identification of different risk and protection factor, paving the way to the development of novel and targeted therapies, and better addressing the health issues of our diverse society.

